# Glomerular filtration rate determined by measuring serum clearance of iohexol in unanesthetized cheetahs (*Acinonyx jubatus*) with comparison to serum symmetric dimethylarginine

**DOI:** 10.1371/journal.pone.0311406

**Published:** 2024-10-01

**Authors:** Nicholas Lordi, Priya Bapodra-Villaverde, Mark Flint

**Affiliations:** 1 One Welfare and Sustainability Center, College of Veterinary Medicine, The Ohio State University, Columbus, Ohio, United States of America; 2 Columbus Zoo and Aquarium, Powell, Ohio, United States of America; National Institutes of Health, UNITED STATES OF AMERICA

## Abstract

One of the more common diseases affecting zoo-managed cheetahs (*Acinonyx jubatus*) is chronic renal disease, which can impact their welfare and ultimately shortens their lifespan. Early diagnosis, for which estimating Glomerular Filtration Rate (GFR) is one such tool, is imperative to help mitigate the negative impacts of this insidious disease. GFR was determined by measuring the serum clearance of iohexol in nine clinically normal, cheetahs managed under human care that presented for voluntary blood collection. A 2-sample iohexol clearance method was performed, along with serum symmetric dimethylarginine (SDMA) determination. SDMA has shown promise in humans, dogs, and cats, as an early biomarker of renal disease. Additionally, the relationship between GFR and SDMA, along with serum creatinine and BUN were analyzed. The mean values for the uncorrected GFR and corrected GFR were 2.08 ± 0.215 mL/min/kg body weight and 1.87 ± 0.173 mL/min/kg body weight, respectively. No significant correlations were observed between GFR, SDMA, serum creatinine, or BUN. Both the uncorrected and corrected iohexol-based GFR values were similar to an inulin-based GFR reference interval determined in zoo managed cheetahs and a reported domestic cat iohexol-based GFR reference interval. Serum SDMA values support previous research suggesting cheetahs have a separate reference interval from domestic cats (0–14 μg/dL). Measuring GFR by the serum clearance of iohexol shows promise as a readily available, cheap, and easily administered clearance marker that can be used in cheetahs trained for voluntary blood collection, thereby avoiding the need for anesthesia.

## Introduction

Among cheetahs (*Acinonyx jubatus*) managed under humane care, renal disease is the most common cause of morbidity and mortality [1]. Glomerulosclerosis and nephrosclerosis are the two most common renal lesions found on necropsy in zoo-managed cheetahs, with the former being identified in over 80% of study populations [1, 2]. The high prevalence of renal disease in captivity is thought to be multi-factorial, with high protein diets, lack of genetic variation, and stress playing a role [2]. Currently, cheetahs are listed as vulnerable by the International Union for the Conservation of Nature’s Red List of Threatened Species, with an estimated 6,517 remaining in the wild and 1,851 being managed under human care per the 2020 International Studbook [3, 4]. Given the declining wild populations of cheetahs, appropriate management and breeding in zoological and conservation facilities can assist in ensuring population stability if used as part of a reintroduction program. A key component in the successful management of the health of zoo-managed populations includes identifying diseases at early onset, and developing appropriate treatments to minimize the deleterious effect of renal disease for individuals whilst also working to reduce the prevalence of disease in zoo-managed populations. Chronic kidney disease (CKD) has been reported as the most frequent cause of death in captive cheetahs between 12 and 16 years of age [5]. Though CKD is incurable, efforts to identify declining renal condition as early as possible can allow for proactive symptomatic treatments that result in improvements in animal health and welfare as well as extending lifespan.

Renal biomarkers such as creatinine and blood urea nitrogen (BUN) are commonly used in the evaluation of renal dysfunction; however, they often only increase after significant loss of renal function [6, 7]. While relatively inexpensive, serum creatinine and BUN screening are not without additional limitations. Extra-renal factors such as muscle mass and dietary protein can alter serum creatinine levels with kidney function being overestimated in cachectic, or low muscled animals, and vice versa [7]. Similarly, dietary protein intake, dehydration, and reduced cardiac output influences BUN levels [8].

Symmetric dimethyl arginine (SDMA) is a novel renal biomarker primarily used in humans, domestic cats and dogs, which can act as a surrogate marker of glomerular filtration rate [7, 9, 10]. In CKD in domestic cats and dogs, SDMA has been shown to increase with only 40% loss of renal function, compared to the required 75% loss of renal function to result in an increase in serum creatinine; allowing earlier detection of subtle pathological changes and subsequent early initiation of medical intervention [6, 10]. Unlike creatinine, SDMA is unaffected by muscle mass and it is mostly filtered and excreted by the kidneys [9–12].

SDMA may have potential use in cheetahs, allowing early renal disease detection and intervention in a species where such advances could help their survival. Although no reference range currently exists for SDMA in cheetahs, the upper limit has been speculated to be similar to 14 μg/dL as with humans, domestic cats, and dogs [13]. Further, SDMA has been shown to increase earlier than serum creatinine and BUN in cheetahs and the IDEXX SDMA immunoassay has been validated in this species [13, 14]. These pieces of data give rise to the potential use of SDMA as an effective renal function marker in cheetahs and warrant investigation.

While SDMA has shown promise as an early biomarker for renal disease in domestic cats and dogs, the gold standard for assessing renal function is to measure glomerular filtration rate [7, 15]. Glomerular filtration rate is defined as the volume of glomerular ultrafiltrate formed in the nephrons of both kidneys per unit time and is directly correlated with functional renal mass [15, 16]. Despite being considered the gold standard, measuring GFR is often not as clinically practical as performing the more routine renal assessment using serum creatinine and BUN, along with a urinalysis to assess urine specific gravity (USG). GFR can be measured using the plasma or renal clearance of an endogenous or exogenous filtration marker. These techniques are often expensive and require multiple timed serum or urine samples [15, 17]. An ideal filtration marker should: 1) not be protein bound and should be freely filtered by the glomerulus, 2) not undergo any metabolism within the body, 3) only be cleared by the kidneys or only have negligible non-renal excretion, 4) not undergo tubular secretion or reabsorption, 5) be non-toxic, and 6) not itself alter GFR following administration [15].

Inulin is considered the gold standard filtration marker for measuring urinary clearance [15]. To measure urinary clearance, the marker must be infused at a continuous rate followed by timed urine collection requiring urinary catheterization and bladder emptying [15]. Given the relatively intensive protocol and lack of availability for practitioners, this method is impractical for routine assessments. Inulin can also be measured via serum clearance [15, 17]. Anesthesia is often required when performing these clearance methods in non-domestic animals due to the intensive, timed sample collections required, and this may impact resulting GFR values [18–20]. Both the urinary and serum clearance of inulin and the resulting glomerular filtration rates have been reported in anesthetized cheetahs along with reference intervals [21, 22]. The earlier study evaluated the urinary clearance of creatinine along with the urinary clearance of inulin and found that the two provided a consistent estimation of GFR in cheetahs [21]. The more recent study measured GFR following a single IV injection of inulin and determined a GFR reference interval for cheetahs [22]. The inulin-based GFR reference interval was similar in both studies [21, 22].

Iohexol is another filtration marker that exhibits the qualities of an ideal filtration marker and has been used for measuring GFR in humans, cats, and dogs [15, 23, 24, 26, 27]. Iohexol (Omnipaque, 300 mg iodine/mL, MWI Animal Health, Whitestown, IN 46075, USA) is a commercially available radiographic contrast agent for intravenous use and does not require urine collection for GFR determination [15, 17, 24]. Disadvantages of the plasma clearance of iohexol include the need for precise serum sampling times as well as the appropriate correction formulas, particularly if using a limited sample method [15, 17, 24–27].

With the potential to test SDMA as a proxy of renal health in zoo managed cheetahs, the aims of this study were to evaluate: 1) whether the plasma clearance of iohexol could be used to measure GFR in unanesthetized, clinically normal cheetahs as a potential stall side test; and 2) the correlation between GFR with serum SDMA, creatinine, and BUN. We hypothesized that GFR results would be similar to existing reference intervals of GFR in domestic cats when iohexol was used as the filtration marker and differ from the cheetah reference intervals for inulin clearance in anesthetized cheetahs [15, 21, 22, 24]. This is the first report of using plasma iohexol clearance in non-domestic felids, and also the first report of GFR measurement in a non-anesthetized non-domestic felid species.

## Materials and methods

### Ethical approval

The study protocol was reviewed and approved by the animal care and use committee (ACUC) at the Columbus Zoo and Aquarium (CZA) in June 2018.

### Iohexol clearance

The serum clearance of iohexol following IV administration of a single bolus was measured in nine cheetahs at the Columbus Zoo and Aquarium. All nine cheetahs were captive-born, hand-reared adults. Two of the cheetahs were males and the remaining seven were females. Their ages ranged from two to seven years and their body weights ranged from 34.6 to 43.7 kg. All animals were housed in indoor-outdoor enclosures at the Columbus Zoo and Aquarium and were fed a commercially prepared meat diet (Nebraska Carnivore or Feline, Nebraska Brand, North Platte, NE 69103, USA).

As part of routine training at the zoo, all nine selected cheetahs were previously trained by the Animal Care staff using positive reinforcement to participate in voluntary blood draws [28, 29]. All cheetahs were deemed healthy prior to being included in the study, with none being suspected for kidney disease based on prior blood work within the previous 12 months.

Prior to measuring iohexol clearance, food was withheld for 12 hours, but free access to water was allowed. The cheetahs were trained to lay in lateral recumbency, and a 23 to 21-gauge needle (SURFLO Winged Infusion Set, Terumo, Somerset, NJ 08873, USA) was inserted into the medial saphenous vein after application of isopropyl alcohol. An initial blood sample was collected into a serum clot activator tube (BD Vacutainer Rapid Serum Tube, BD, Mississauga, ON L5N 0B3, CA) as well as an EDTA collection tube (BD Vacutainer K2EDTA Tube, BD, Mississauga, ON L5N 0B3, CA) for serum biochemistry and complete blood count analysis. After collecting the initial blood sample, a single bolus of iohexol at 0.5 mL/kg (Omnipaque iohexol, 300 mg iodine/mL, MWI Animal Health, Whitestown, IN 46075, USA) was administered through the same butterfly needle used for blood draw slowly over 60 seconds, as has been described in domestic cats [15, 24]. Doses ranged from 18–22 milliliters (5.4–6.6 g iodine) based on animal weights. The completion of iohexol injection represented time 0. The butterfly needle was then flushed with sterile 0.9% saline and removed. For the remaining sample collections at Times 120 minutes and 240 minutes post-iohexol injection, the cheetahs were housed in the indoor portion of their enclosures.

In order to determine GFR using a two-sample iohexol technique, blood samples (3–4 mLs) were collected into serum clot activator tubes 120 and 240 minutes after iohexol administration. Samples were allowed to clot and then centrifuged at 2500 rpm for ten minutes. A minimum of 1.2 mLs of serum was then collected and transferred to a 3 mL plastic vial labeled with the exact sampling time and animal ID. Samples were stored in a refrigerator until shipment the same day. Blood samples were shipped in insulated mailers (Michigan State University, Lansing, MI 48910, USA) with an ice pack to the Michigan State University Veterinary Diagnostic Laboratory (MSU-VDL) for determination of the iohexol concentration. MSU-VDL measured the serum iohexol clearance using Inductively Coupled Plasma-Mass Spectrometry (ICP/MS).

### Calculation of GFR

GFR measurements were provided by the Michigan State University Veterinary Diagnostic Laboratory and were considered uncorrected due to being calculated from only two iohexol clearance samples rather than the standard three samples. A correction formula utilized in domestic cats during cases of limited iohexol clearance sampling was applied to the uncorrected values: Corrected Clearance = (1.036 x GFR_uncorrected_− 0.062 x GFR_uncorrected_^2^ [15, 23]. The formula was applied due to the uncorrected GFR values likely overestimating actual GFR when using the one compartment model assumption [15, 24]. This assumption was elected for the values provided by MSU-VDL because in theory, it requires only two samples [15].

### Serum SDMA, creatinine, and BUN concentration

A red-top tube containing serum and an EDTA tube containing whole blood collected just prior to the iohexol bolus administration were submitted on ice to IDEXX Laboratories (Worthington/Columbus, Worthington, OH 43085, USA) for determination of SDMA, creatinine, BUN, along with standard serum chemistry panel and complete blood counts. SDMA concentrations were determined using a commercially available high-throughput immunoassay (IDEXX SDMA Test, IDEXX Laboratories, Westbrook, ME 04092, USA), which has since been validated in cheetahs [13, 14].

### Statistical analysis

Descriptive statistics were calculated for GFR, serum SDMA, serum creatinine, BUN, and age. The Shapiro-Wilk test was used to evaluate whether data were normally distributed. Spearman’s rank correlation was used to measure the correlation between variables. Grubb’s test was used to evaluate the data for outliers. All calculations were performed using STATA BE (Version 17.0, StataCorp, 4905 Lakeway Drive, College Station, Texas 77845 USA), Microsoft Office Excel (2011), and R (R Version 4.2.3, R Studio Version 1.1.463). P-values ≤ to 0.05 were considered significant.

## Results

**Fig 1:**
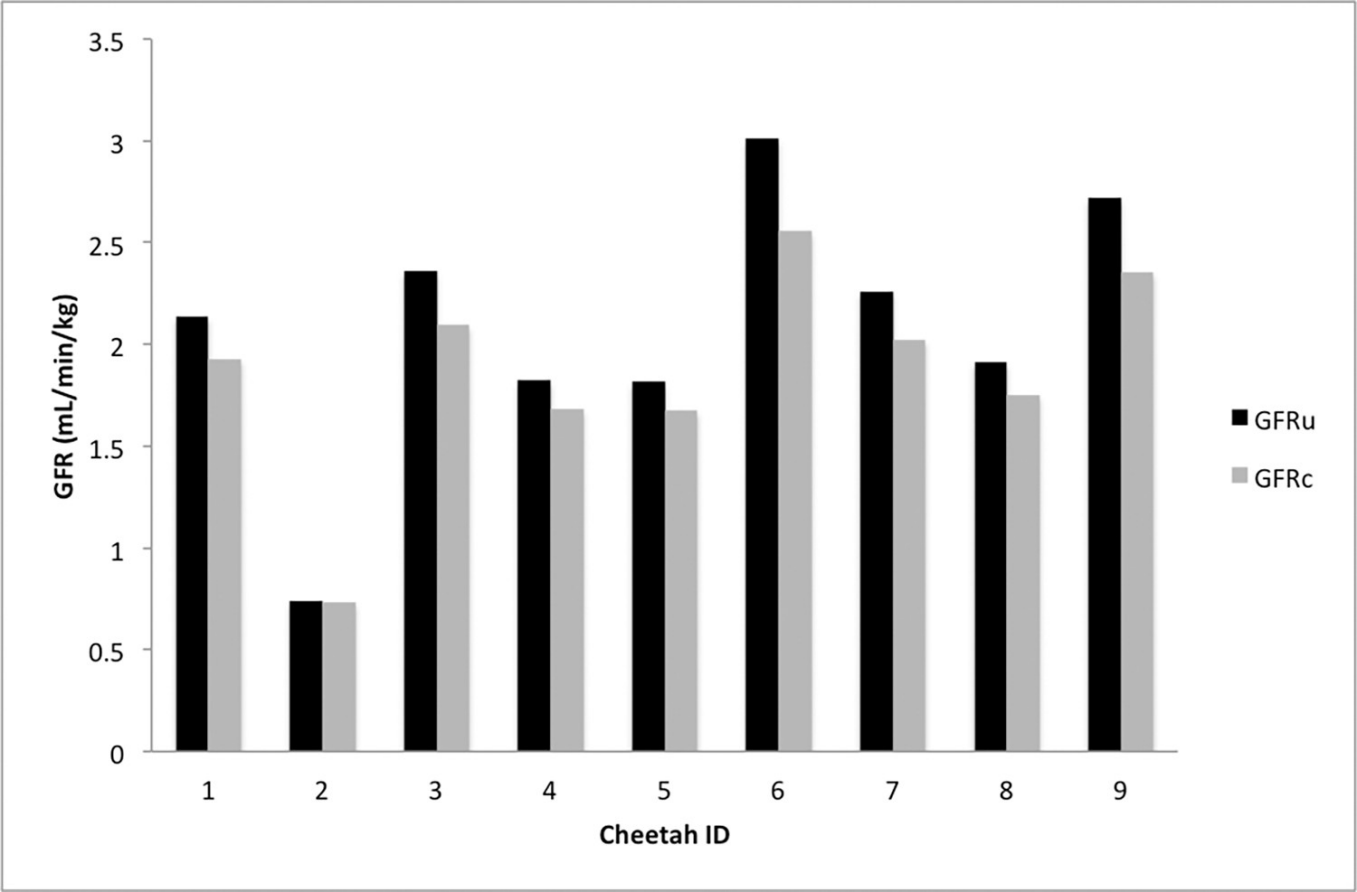
Glomerular filtration rate among cheetahs (*Acinonyx jubatus*) in the study population. The uncorrected GFR (GFRu) and corrected GFR (GFRc) of nine, clinically normal cheetahs are displayed. GFRc was calculated by applying the domestic cat formula for limited sampling when using iohexol to uncorrected GFR values [15]. GFRu and GFRc have an extremely strong, positive correlation (r = 0.998; ρ<0.001). The mean ± SD for the uncorrected GFR was 2.08 ± 0.215 mL/min/kg (range: 0.74–3.009 mL/min/kg). The mean ± SD for corrected GFR was 1.87 ± 0.173 mL/min/kg (range: 0.732–2.556 mL/min/kg).

Uncorrected and corrected GFR values were evaluated in nine cheetahs (2M7F) (Fig 1). A normal distribution was observed for creatinine (ρ = 0.389), uncorrected GFR (ρ = 0.528), and corrected GFR (ρ = 0.296) after applying the Shapiro-Wilk test to the data set. The observed mean ± SD for the uncorrected GFR was 2.08 ± 0.215 ml/min/kg (Table 1). The minimum value for the uncorrected GFR was 0.74 ml/min/kg, while the maximum value was 3.009 ml/min/kg. After applying the domestic cat correction formula [15] for GFR measured using the serum clearance of iohexol, the observed mean ± SD for corrected GFR was 1.87 ± 0.173 ml/min/kg (Table 2). The minimum value for corrected GFR was 0.732 ml/min/kg, while the maximum value was 2.556 ml/min/kg. An extremely strong, positive correlation was observed between uncorrected and corrected GFR (r = 0.998; ρ<0.001). Iohexol clearance, and the resulting GFR, could not be determined using the slope intercept method and linear regression analysis, as has been done in other studies, due to the limited sampling method chosen for this study [15, 22, 24]. The Grubb’s test was used to evaluate for potential outliers. No outliers were detected throughout the data set, including the minimum uncorrected and corrected GFR values observed for Cheetah 2 (ρ = 0.117, and ρ = 0.064, respectively). No adverse effects were observed or reported in any of the cheetahs following the IV administration of iohexol.

**Table 1:** Descriptive statistics for age, GFR, serum creatinine concentration, serum BUN concentration, and serum SDMA concentration in 9 clinically normal cheetahs (*Acinonyx jubatus*).

	Mean	Median	1 Standard Deviation	Range	95% CI for mean
**Age (years)**	3.55	4	1.74	2–7	2.21–4.89
**GFR (mL/min/kg)** ^**a**^	2.08	2.14	0.215	0.74–3.009	1.59–2.58
**GFR (mL/min/kg)** ^**b**^	1.87	1.93	0.173	0.732–2.556	1.47–2.27
**SDMA (μg/dL)**	12.67	12	2.78	10–16	10.53–14.81
**Creatinine (mg/dL)**	2.51	2.5	0.40	2.0–3.1	2.20–2.82
**BUN (mg/dL)**	38.55	38	6.17	32–53	33.82–42.23

GFR, glomerular filtration rate; SDMA, symmetric dimethylarginine; BUN, blood urea nitrogen.

^a^Uncorrected GFR provided by Michigan State University Veterinary Diagnostic Laboratory (MSU-VDL).

^b^Corrected GFR using domestic cat formula for iohexol clearance measurements: Corrected Clearance = (1.036 x GFR_uncorrected_− 0.062 x GFR_uncorrected_^2^. [15]

**Table 2:** Collective measurements for nine unanesthetized, clinically normal cheetahs (*Acinonyx jubatus*).

Cheetah ID	Sex	Age (yrs)	Weight (kg)	SDMA (μg/dL)	Creatinine (mg/dL)	BUN (mg/dL)	GFR (mL/min/kg)^a^	GFR (mL/min/kg)^b^
1	F	5	41.1	10	2.1	32	2.14	1.93
2	F	7	36.9	15	3.1	42	0.74	0.732
3	F	4	36.2	15	2.9	36	2.36	2.095
4	M	2	42.3	12	3	53	1.823	1.682
5	F	2	36.7	10	2.2	38	1.815	1.676
6	F	2	34.6	10	2	39	3.009	2.556
7	M	4	43.7	10	2.5	35	2.26	2.024
8	F	4	43.2	16	2.5	34	1.911	1.753
9	F	2	36	16	2.3	38	2.718	2.357

ID, identification; SDMA, symmetric dimethylarginine; BUN, blood urea nitrogen; GFR, glomerular filtration rate.

^a^Uncorrected GFR provided by Michigan State University Veterinary Diagnostic Laboratory (MSU-VDL).

^b^Corrected GFR using domestic cat formula for iohexol clearance measurements: Corrected Clearance = (1.036 x GFR_uncorrected_− 0.062 x GFR_uncorrected_^2^ [15].

No significant correlations were observed between the uncorrected GFR and SDMA (r = -0.018, ρ = 0.9642), serum creatinine (r = -0.519; ρ = 0.152), or BUN (r = -0.218; ρ = 0.5739). There were also no significant correlations observed between the corrected GFR and SDMA (r = -0.18; ρ = 0.964), serum creatinine (r = -0.519; ρ = 0.1524), or BUN (r = -0.218; ρ = 0.5739).

**Fig 2:**
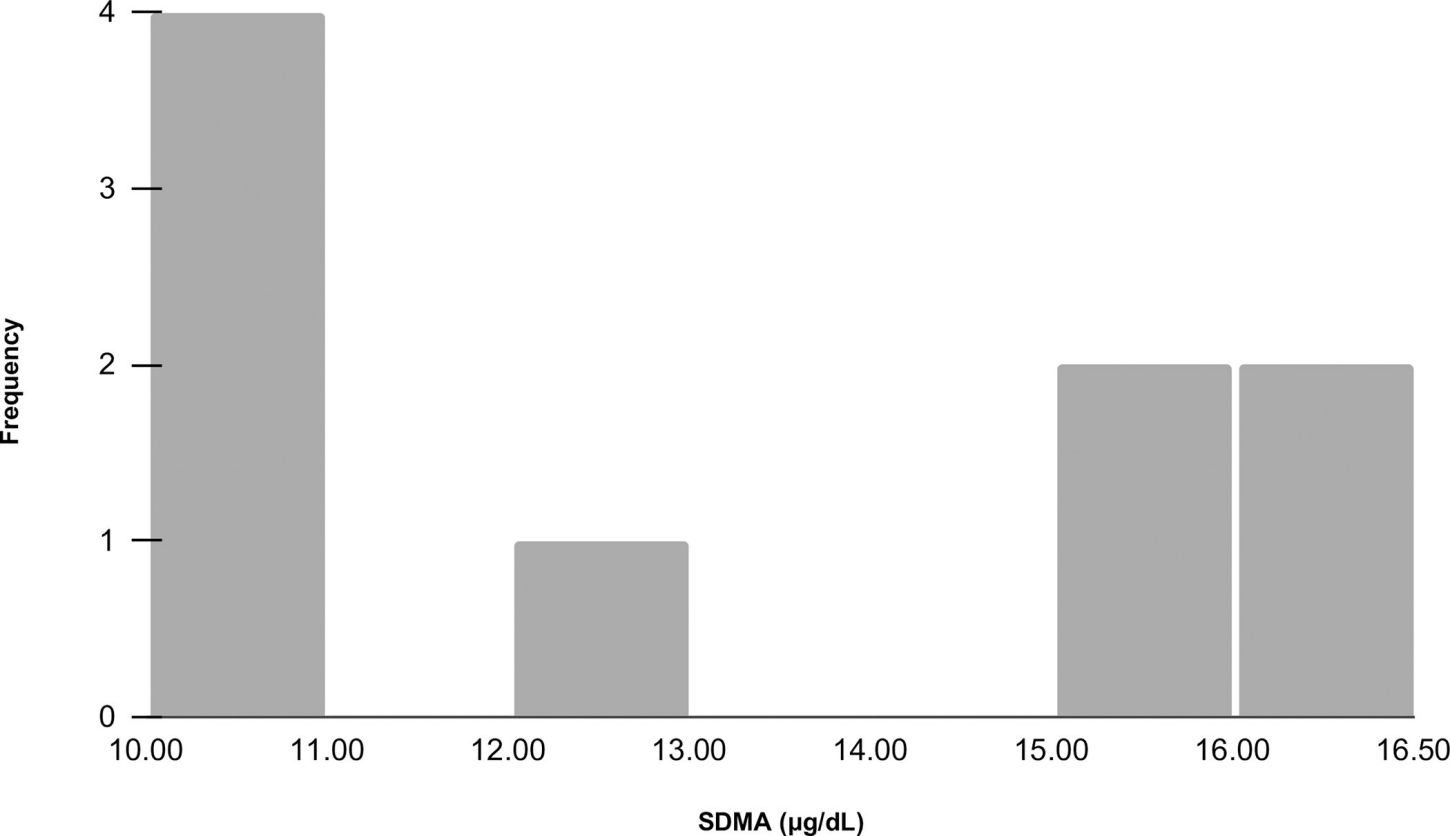
Histogram of serum SDMA concentration in nine, clinically normal cheetahs (*Acinonyx jubatus*). Serum was collected once from each unanesthetized cheetah and SDMA (symmetric dimethylarginine) levels were provided by a diagnostic laboratory. SDMA ranged from 10–16 μg/dL. The mean ± SD for SDMA was 12 ± 2.78 μg/dL. A normal distribution was not observed among the study population.

SDMA was not normally distributed among the study population (ρ = 0.011, Fig 2). No significant correlations were observed between serum SDMA concentration and BUN (r = 0.0329; ρ = 0.0.829) or serum creatinine (r = 0.503; ρ = 0.168). There were no significant correlations observed between age and any of the listed variables (Table 2).

## Discussion

The aims of this study were met. We determined that plasma clearance of iohexol could be used to measure GFR in un-anesthetized, clinically normal cheetahs, and we determined the correlation between GFR with serum SDMA, creatinine, and BUN was poor. To our knowledge, this study represents the first time that GFR values were determined from the serum clearance of a single IV injection of iohexol, as well as the first time that GFR had been measured in un-anesthetized non-domestic felids. Both of these findings are of value to the clinician working with these animals. Our hypothesis was partially upheld. Corrected GFR values were comparable with those published for domestic cats and cheetahs [15, 22, 24].

The minimum and maximum Cheetah GFR values, pre-correction, were 0.74 and 3.01 mL/min/kg, respectively. There are various iohexol-based GFR reference intervals for healthy, domestic cats. We used MSU-VDL’s 1.15–2.73 mL/min/kg range. The inulin based GFR reference interval for cheetahs was found to be 0.84 to 2.37 mL/min/kg [22]. While the majority of our values fell within both existing reference intervals, variances may be explained by physiologic differences between domestic and non-domestic felids, the use of anesthesia during sample collection in other trials, the use of different GFR clearance markers, and the 2-sample iohexol clearance used in this study [19, 20, 22, 30–33].

Among our study population, Cheetah 2 had low GFR when compared to the other animals sampled (uncorrected GFR: 0.74 mL/min/kg, corrected GFR: 0.732 mL/min/kg), slightly lower than the inulin-based GFR range published for cheetahs [22]. Statistical analysis did not support outlier status for the uncorrected or corrected GFR values observed for Cheetah 2 (ρ = 0.117, and ρ = 0.064, respectively). Cheetah 2 was the oldest cheetah in the study (7 years) but had no previous history of renal compromise and no extravasation of the IV iohexol was appreciated during administration, both of which may result in a lower measurement. It is generally thought that GFR decreases with age [31, 32, 34–36], however, no significant correlation was observed between GFR and age in the present study (r = -0.337; ρ = 0.375) and no evidence of any renal disease was detected in any of the animals at the time of the study or the two years following.

Although measuring GFR using iohexol was the primary aim of the study, we also evaluated SDMA and its relationship to GFR, serum creatinine, and BUN. We did not find any correlation between serum SDMA and GFR, serum creatinine, or BUN. These findings were similar to the inulin-based GFR study in cheetahs [22]. Other studies involving cheetahs and renal biomarkers have observed significant positive correlations between SDMA and serum creatinine, however, those animals were already known to have chronic renal disease unlike our study population [13, 14, 37]. SDMA was not compared to GFR in any of the studies. A limitation regarding SDMA in our study was the lack of an existing reference interval for cheetahs. At the time of the study, it had been assumed that the normal reference interval for cheetahs had an upper limit of 14 μg/dL, which was similar to what had been observed in humans, dogs, and domestic cats [13, 38]. Given that our sample size included SDMA values of 15 and 16 μg/dL in young, middle aged, and older cheetahs, it is likely that cheetahs have a reference interval higher than noted for domestic cats. While SDMA has shown promise in both human and domestic animal medicine, further evaluation with GFR, as well as the establishment of a reference interval, are warranted to determine the biomarker’s usefulness as an early indicator of renal disease in non-domestic felids.

Limitations of our study include small sample size (n = 9), evaluating only clinically healthy cheetahs with no evidence of renal disease, and the 2-sample serum iohexol clearance measurement not being validated in non-domestic felids. Per the American Society for Veterinary Clinical Pathology reference interval guidelines, a minimum of 120 samples is recommended to determine reference ranges by nonparametric methods with 90% confidence intervals and reference intervals should not be calculated for samples less than 20 [39]. Our sample size did not allow us to create a reference interval for GFR when using the serum clearance of iohexol. Given the desire to measure GFR without the need for anesthesia, all nine cheetahs were trained for voluntary blood collection and were considered to be free of clinical renal disease. Renal disease was not suspected in any of the nine cheetahs that participated based on prior physical exam and recent blood work. Lastly, one of the primary limitations was that GFR measurements using iohexol have not been validated in cheetahs. In an ideal setting, four to eight serums samples at various time points would be collected, and the serum clearance of iohexol would then be used to calculate GFR [15, 24]. With our goal of not only evaluating GFR in cheetahs using iohexol, but also measuring GFR in a non-anesthetized cheetah as a possible patient side renal assessment, we knew a limited sampling method would be more appropriate. In order to establish consistency in sampling across the nine cheetahs, a 2-sample serum collection seemed the most appropriate not only for the cheetahs with variable years of blood collection training experience, but also for the animal care and animal health staff involved. Although the domestic cat correction formula for iohexol-based GFR has not been validated in cheetahs, its application to iohexol-based GFR values in non-domestic felids should be considered as a potential correction for assumptions, particularly in cases of limited sampling.

Our study measured the first ever GFR values in an awake, non-domestic felid species, via the serum clearance of iohexol. Our uncorrected GFR values were similar to the range of the inulin-based GFR reference interval for cheetahs and the domestic cat reference interval provided by MSU-VDL. After applying the domestic cat correction formula, values aligned more closely with previously published cheetah reference intervals. Further investigations into iohexol-based GFR measurements are warranted, ideally attempting to collect a minimum of three serum samples. Iohexol offers the potential for a quick and easy patient side collection to determine GFR without the need for anesthesia in cheetahs trained for voluntary blood collection. Pending further investigation of iohexol clearance in cheetahs, there is the potential for requiring only a single serum sample collection to determine GFR as has already been validated in domestic cats [24]. Additionally, SDMA should be incorporated into other non-domestic felid studies evaluating GFR in order to better characterize the biomarker’s usefulness. It would also be of benefit in future GFR studies to incorporate additional renal diagnostics, such as urinalysis and renal ultrasound, to provide a more comprehensive renal assessment. Our study provides those caring for non-domestic felids, particularly cheetahs, the potential for incorporating GFR based on serum iohexol clearance into clinical renal evaluations. Though further studies are needed, including assessing cheetahs with known renal dysfunction, iohexol has shown potential as a safe, inexpensive, commercially available clearance marker for use in the gold standard assessment of renal function, GFR.
